# USING PROJECT ECHO FOR GERIATRIC TRAINING IN RURAL COMMUNITIES

**DOI:** 10.1093/geroni/igae098.0067

**Published:** 2024-12-31

**Authors:** Sarah Ross, Susanna Luk-Jones, Sara Murphy, Matthew Biggerstaff, Jennifer Severance

**Affiliations:** University of North Texas Health Science Center, Fort Worth, Texas, United States; .University of North Texas Health Science Center at Fort Worth, Fort Worth, Texas, United States; University of North Texas Health Science Center, Fort Worth, Texas, United States; University of North Texas Health Science Center, Fort Worth, Texas, United States; University of North Texas Health Science Center, Fort Worth, Texas, United States

## Abstract

Expanding geriatric education and training to health care teams using the Project ECHO (Extension for Community Healthcare Outcomes) model can improve access to quality geriatric care in rural communities. The UNTHSC Center for Older Adults applied the Project ECHO model to deliver interactive videoconferencing sessions and connect interprofessional teams of geriatric specialists with primary care providers and the nursing home workforce. Recruitment included social media marketing and mailing campaigns to target rural and other underserved communities in Texas and the surrounding regions. Training was offered as weekly, bi-weekly, or monthly ECHO sessions featuring specialist-led didactic presentations followed by interactive breakout discussions across a variety of geriatric topics in dementia care, workforce considerations, and age-friendly healthcare. Participants completed post-session surveys to track changes in geriatric care knowledge, skills, and abilities using a five-point Likert scale. From September 2019 to September 2022, a total of 157 ECHO sessions were conducted with 437 unique participants from 77 counties in 14 states. Of these 77 counties, 23 (29.9%) were rural. The most common job titles were nursing (36.2%), administration and leadership (24.9%), physician (12.1%) and student (7.9%). Out of the 437 unique attendees, 158 (36.2%) participated in at least 25% of the sessions. Participants completed post-session surveys to track changes in geriatric care knowledge, skills, and abilities using a five-point Likert scale, with positive scores on all measures.

